# Temporally Targeted Interactions With Pathologic Oscillations as Therapeutical Targets in Epilepsy and Beyond

**DOI:** 10.3389/fncir.2021.784085

**Published:** 2021-12-08

**Authors:** Tamás Földi, Magor L. Lőrincz, Antal Berényi

**Affiliations:** ^1^MTA-SZTE “Momentum” Oscillatory Neuronal Networks Research Group, Department of Physiology, University of Szeged, Szeged, Hungary; ^2^Neurocybernetics Excellence Center, University of Szeged, Szeged, Hungary; ^3^HCEMM-USZ Magnetotherapeutics Research Group, University of Szeged, Szeged, Hungary; ^4^Child and Adolescent Psychiatry, Department of the Child Health Center, University of Szeged, Szeged, Hungary; ^5^Department of Physiology, Anatomy and Neuroscience, Faculty of Sciences University of Szeged, Szeged, Hungary; ^6^Neuroscience Division, Cardiff University, Cardiff, United Kingdom; ^7^Neuroscience Institute, New York University, New York, NY, United States

**Keywords:** oscillation, oscillopathy, brain stimulation, closed-loop, epilepsy

## Abstract

Self-organized neuronal oscillations rely on precisely orchestrated ensemble activity in reverberating neuronal networks. Chronic, non-malignant disorders of the brain are often coupled to pathological neuronal activity patterns. In addition to the characteristic behavioral symptoms, these disturbances are giving rise to both transient and persistent changes of various brain rhythms. Increasing evidence support the causal role of these “oscillopathies” in the phenotypic emergence of the disease symptoms, identifying neuronal network oscillations as potential therapeutic targets. While the kinetics of pharmacological therapy is not suitable to compensate the disease related fine-scale disturbances of network oscillations, external biophysical modalities (e.g., electrical stimulation) can alter spike timing in a temporally precise manner. These perturbations can warp rhythmic oscillatory patterns via resonance or entrainment. Properly timed phasic stimuli can even switch between the stable states of networks acting as multistable oscillators, substantially changing the emergent oscillatory patterns. Novel transcranial electric stimulation (TES) approaches offer more reliable neuronal control by allowing higher intensities with tolerable side-effect profiles. This precise temporal steerability combined with the non- or minimally invasive nature of these novel TES interventions make them promising therapeutic candidates for functional disorders of the brain. Here we review the key experimental findings and theoretical background concerning various pathological aspects of neuronal network activity leading to the generation of epileptic seizures. The conceptual and practical state of the art of temporally targeted brain stimulation is discussed focusing on the prevention and early termination of epileptic seizures.

## Introduction: Physiological and Pathological Brain Oscillations

Neuronal oscillations are rhythmic neuronal activities that synchronize different operations within and across neuronal networks (Buzsáki, 2009). The broadband neural signals recorded as the potential fluctuations of the extracellular electrical field can be analyzed to extract signals of various frequency bands. On one hand, the low frequency local field potentials (LFPs), representing the summed transmembrane currents from numerous neurons and on the other hand fast transients (lasting less than a millisecond) represent action potentials (APs) (Buzsáki et al., 2012). Action potentials and LFPs present in raw recording traces can be decomposed by Fourier transformation into various frequency bands on a spectrogram with LFPs and action potentials inhabiting distinct frequency bands (in general < 250 Hz for physiological LFPs, and > 250 Hz for single-unit action potentials). These can be discriminated by applying analog or digital filtering to preferentially pass signals in lower or higher frequency bands, respectively (Hong and Lieber, 2019). The primary origin of neuronal oscillations is the periodical synchronization of synaptic potentials influenced by the periodical fluctuation of excitability in clusters of neurons. The rhythmicity hail from network structures comprising a variety of distinct cell types and population activities (Buzsáki and Watson, 2012). In addition to synaptic activity extracellular field potentials can influence the neuronal membrane potential via ephaptic coupling resulting in altered neuronal firing (Anastassiou et al., 2011). Hence, oscillations and neuronal activities in the brain are cohesive and self-arranged. Oscillations offer an effective potential mechanism for integrating the activity of single neurons toward microcircuits and extensive functional neuronal networks facilitating interregional communication and information processing (Engel et al., 2001; Buzsáki and Draguhn, 2004). Oscillations indicate applicable network conditions, impact neuronal population operations in the network; and constitute the dynamics of macroscopic neuronal networks intimately linked to the behavioral phenotypes on several levels of biological systems (Leuchter et al., 2015). Therefore, the concurrence of altered pathological oscillations and abnormal behavioral phenotypes in neurological and psychiatric diseases is unsurprising; these disorders can be regarded as “Oscillopathies” (Mathalon and Sohal, 2015). Pathologic oscillations represent multiple interactions and a causal relationship with abnormal brain states and functions, respectively. Thus, the pathological oscillations constitute a potential target for therapeutic intervention by applying the recently developed time-and space-targeted brain stimulation technologies (Berényi et al., 2012; Vöröslakos et al., 2018), an approach termed “Oscillotherapeutics” (Takeuchi and Berényi, 2020).

## Epilepsy

Epilepsy is a typical oscillopathy, where the altered neuronal activity results in altered oscillations leading to impaired brain functions. Epilepsy is a common neurological disease characterized by a chronic susceptibility to develop recurring epileptic seizures (Fisher et al., 2014). An epileptic seizure is a temporal behavioral alteration that can mediate objective, noticeable (e.g., muscular contractions) or subjective, covert manifestations (e.g., loss of consciousness). These alterations are presumably generated by hypersynchronous neural activities in various brain networks. Electroencephalography (EEG) is a non-invasive method which, measures the electrical activity of large, synchronously firing populations of neurons with electrodes placed on the scalp. The synchronized neural activity is evident in EEG or intracerebral LFP records during seizures (termed ictal periods) and will lead to specific behavioral manifestations, such as tonic and clonic convulsions among others. Effective pharmacotherapy and neurosurgical intervention in epileptic patients can systematically decrease the occurrence rate of electrographic and behavioral seizures (Glauser et al., 2006). In addition, time-targeted intervention of the abnormal neural oscillations characterizing preictal or ictal states can curtail their behavioral manifestation (Morrell, 2011) indicating a causal association between pathological oscillations and the symptoms of epilepsy.

## Clinical Significance

### The Role of the Hippocampus in Temporal Lobe Epilepsy

Temporal lobe epilepsy (TLE) is frequently pharmaco-resistant and its uncontrolled generalized seizures increase the risk of sudden unexpected death in epilepsy (Bone et al., 2012; Massey et al., 2014). Surgical resection of the seizure focus is irreversible, massively invasive and can frequently lead to cognitive disorders (Hamberger and Drake, 2006). Furthermore, its implementation in patients with ambiguous or multifocal bilateral TLE is not feasible (Berg et al., 2003; Holmes et al., 2003). Multiple studies have shown altered functional networks in TLE, including those explicitly involving the seizure focus in the hippocampus (Bettus et al., 2010; Englot et al., 2016). Functional network alterations have been reported to relate to neurocognitive disability and surgical treatment outcome (Holmes et al., 2014; Morgan et al., 2017, 2020b). On the other hand, it is well known that physiological hippocampal function requires a complex and particular spatiotemporal activation system (Cooper and Ritchey, 2019). Even in the case of high frequency oscillations the phase coherence of these signals fluctuates on the order of seconds. A recent study showed that increases in the variance of signal fluctuations occurring at the hippocampal seizure focus in patients with TLE might contribute to disruptions in physiological functional connectivity (FC) network dynamics that contribute to decreases in static hippocampal FC on fMRI scans (Morgan et al., 2020a).

We have previously shown that closed-loop electrical stimulation of the medial septum can quickly terminate intrahippocampal seizures while also suppressing their secondary generalization in a rat kindling model (Takeuchi et al., 2021). Still, as was the case for DBS, further translational research is required to employ the transcranial techniques.

### Absence Epilepsy

TES has already been proven to successfully reduce the duration of spike-and-wave discharges (the electrographic hallmarks of human absence epilepsy) in a rodent model of generalized epilepsy (Berényi et al., 2012). Its efficient clinical application will rely on closed-loop feedback stimulation of the target circuits, as their modulation can interfere with the emerging pathological pattern (Berényi et al., 2012; Kozák and Berényi, 2017). In addition, closed-loop seizure suppression using TES can remain effective for long periods (i.e., months) (Kozák and Berényi, 2017).

### Other Neuropsychiatric Disorders

Many neurological and psychiatric disorders are related to clinically discernible, altered brain dynamics. These pathological oscillations may be a target for therapeutic intervention for the disorders using time-and space-targeted brain stimulation technologies.

Major depressive disorder (MDD) is a common and chronic psychiatric disorder characterized by excessive feelings of sadness and low mood (American Psychiatric Association, 2013). The most relevant oscillopathic features of MDD are: increased alpha-band (8–13 Hz) activity in the temporo-parietal area, elevated frontal theta-band (4–7 Hz) activity, alpha frontal asymmetry (left hemispheric hypoactivity and right hemispheric hyperactivity expressed as theta, alpha and beta band activities) and decreased gamma band activity in the neocortex (Baskaran et al., 2012; Eidelman-Rothman et al., 2016; Fitzgerald and Watson, 2018). These features relate to MDD symptoms and predict the efficiency of pharmacological treatment and electroconvulsive therapy. In addition, a causal relationship between oscillopathies and symptoms of (major) depression may exist. Indeed, restoration of the frontal alpha symmetry using anodal tDCS on the dorsolateral prefrontal cortex (DLPFC) (Loo et al., 2012) and neurofeedback improved the symptoms of depression (Mennella et al., 2017). These pathological oscillations can be targeted using pharmacological and electrical stimulation methods in combination with cognitive (behavioral) methods to alleviate depression symptoms (Leuchter et al., 2015).

Posttraumatic stress disorder (PTSD) is a widespread neuropsychiatric disorder with a high burden of disease. Primary symptoms include anxiety, cognitive impairments, mood changes and consistent avoidance of trauma-related stimuli (American Psychiatric Association, 2013). The panic, fear, and sympathetic response to the trigger stimulus results from altered activity in the amygdala (Cisler et al., 2015). Deficiency of fear extinction is also a salient feature of PTSD. Closed-loop intervention can rely on real-time correlates of neural network activation and various symptoms. In PTSD patients hyperactivity characterizes resting magnetoencephalography (MEG) recordings of the amygdala, the hippocampus, and the insular cortex (Huang et al., 2014). Altered activity also characterizes EEG recordings of PSTD patients i.e., intrinsic sensory hyperactivity in the visual cortex (suppressed alpha power) and decreased alpha power-mediated inhibition to the frontal cortex (Clancy et al., 2017). Closed-loop stimulation of the amygdala can reduce dysregulated amygdala responses (Stidd et al., 2013; Koek et al., 2016).

## Network Models of Pathological Patterns and What Can We Conclude From Them

### Cellular Activity Underlying Seizures and Epilepsy

Generally, epilepsy is thought to root in neuronal hyperexcitability (Fisher et al., 2005). Several underlying mechanisms have been proposed based mainly on the results obtained from animal models, including impaired inhibition (Bekenstein and Lothman, 1993) or a change in excitatory neurons’ intrinsic conductances, leading to an overall increase of network output and synchrony (Avoli et al., 2005). Monitoring the activity of single neurons in the human brain can reveal important aspects of brain function. However, it is more challenging to identify the role of individual neurons in epilepsy primarily because of the sparseness of seizures and the technical limitations of long-term single-unit recordings. There are far more studies concerning the interictal neuronal activity in human epilepsy, which revealed significant differences between affected and non-affected areas, including differences in firing rates, bursting and synchrony (Staba et al., 2002; Gast et al., 2016). The few successful attempts in which the ictal activity of single neocortical or hippocampal neurons was recorded revealed surprising results. Synchronous firing of neighboring neurons was rarely seen except at the onset of ictal events (Wyler et al., 1982). Seizures can provide intense and synchronous, yet sparse and heterogeneous activation (Bower et al., 2012). Besides this surprising heterogeneity, a general lack of hypersynchrony suggests that specific interactions among subsets of neurons initiate seizures (Truccolo et al., 2014; Lambrecq et al., 2017). On the other hand, seizure termination is characterized by a relatively homogeneous suppression of firing (Truccolo et al., 2014).

## Seizure Activity and Coupled Oscillations

A recent study showed that in the intact isolated mouse hippocampus, a paroxysmal activity can spread through the hippocampus following seizure onset, both from a focal stimulation locus or if low magnesium was applied locally to either longitudinal ends of the preparation (Derchansky et al., 2006). Bursts of activity within a seizure can become bidirectional, with frequency specific propagation patterns. In the low magnesium model, independent bidirectional activity was observed on both sides when the isolated intact hippocampus was severed along the septotemporal axis. These activities are in agreement with the function of coupled neuronal network oscillatory systems. Local coherence and ictal activity transfer was assessed in the recordings from intra-hippocampal depth electrodes implanted in epileptic patients being evaluated for possible resective surgery (Duckrow and Spencer, 1992). It was found that although ictal neural rhythmicity involves a temporal interaction between brain regions, the maintenance of this interaction is not essential for persistent seizure activity. These findings are in line with the idea of seizures being the manifestation of a multistate network of oscillatory systems showing various degrees of coupling and uncoupling.

### ”Coupled Oscillators” Model of Hyperexcitable Neuroglial Networks

Epilepsy is a dynamic disorder showing characteristics of neural networks with the incidence of at least two states, known as interictal and ictal activities (Lopes da Silva et al., 2003). The brain can be considered as a system of coupled oscillatory (multistate) units, and epilepsy a pathological expression of this system. The advantage of using a coupled oscillator approximation to model epilepsy is its ability to effectively model intermittent phenomena in epileptic brain networks (Zalay and Bardakjian, 2009). An attractor state is a transiently self-sustaining state (Meindertsma and Steenbeek, 2012). Unlike the multistate bistable attractor technique, intermittence corresponds to ictal events integral in the interictal attractor (or state) and doesn’t require system noise for state transition (in these models, the critical mechanism for transitions to and from epileptic seizures is the existence of multiple attractors). A model that exploits this approach has been used to analyze different pathways leading to hyperexcitability and recommended a critical role for astrocytes and microglia in generating spontaneous epileptiform discharges (SEDs) (Farah et al., 2019). This model was built on the concept of coupled Cognitive Rhythm Generators (CRGs). The CRG is a mesoscopic mathematical modeling frame, used to model different physiological phenomena, such as directional selectivity, phase preference and phase precession (Zalay and Bardakjian, 2009). In addition, a network of four coupled CRGs has been used to model hippocampal neurons and generate SEDs (Zalay et al., 2010). This oscillator approximation might be a clock with a universal rhythm or a labile clock, where the oscillator is only active when the input is higher than a set threshold. The model included 16 CRGs organized into four subgroups with excitatory pyramidal cells, inhibitory interneurons, microglia and astrocytes. Pyramidal cell CRGs exhibited constant rhythmicity with intrinsic frequencies in the theta range (McNaughton et al., 1983), similar to results obtained from experimental recordings (Bezaire et al., 2016). Bursting activity of interneurons was characterized by labile clock behavior in the ripple HFO frequency range (80–250 Hz) (Sik et al., 1995), as is seen in experimental seizure-like events (Zalay et al., 2010). Microglial CRGs were modeled as a clock ring device with slow oscillations (0.2–0.5 Hz) (Wake et al., 2009). Lastly, the activity of astrocytes was characterized by labile clock behavior spanning the 1–4 Hz frequency range (Amzica and Steriade, 2000).

Astrocytes can regulate the excitability of adjacent neuronal synapses (Perea et al., 2009) and astrocytic dysfunction is related to several neurological disorders including epilepsy (Seifert et al., 2010). Earlier modeling studies highlighted the importance of glial function in K^+^ homeostasis in hyperexcitability, suggesting glial function can act as a biomarker for epilepsy (Grigorovsky and Bardakjian, 2018). The increase in neuron-astrocyte coupling provoked a higher occurrence of SEDs, coherent with studies indicating that the release of specific gliotransmitters by astrocytes can predispose neuronal circuits to seizures. In contrast, the magnitude of neuron-microglia coupling was negatively correlated to hyperexcitability, with less SEDs of shorter duration appearing as the microglia-neuron coupling increased (Fellin et al., 2004; Carmignoto and Haydon, 2012). These latter modeling approaches are also consistent with experimental results showing that microglia can preferentially connect to hyperactive neurons, reduce their EPSC rate and down-regulating their activity (Li et al., 2012; Ji et al., 2013). Manipulating certain microglial functions is also related to the occurrence of seizures (Derecki et al., 2012; Eyo et al., 2014).

## Multistate and Bistable Network Models

### Seizure Dynamics: Initiation, Development, and Termination

Epilepsy is a network malfunction described by bistable or multistable oscillatory states (e.g., interictal and ictal states) and their dynamic alternations. To investigate whether this multistate bistable approach can capture seizure dynamics, a divided system of bistable neural units built on an analytic, non-linear complex model was used (Izhikevich, 2001; Kalitzin et al., 2011). Depending on various parameters, this model is able to represent steady-state dynamics, limit cycle dynamics, or both. Ad absurdum, the model could be mentioned as a bistable unit. Based on the primary conditions, the bistable unit can either be in its steady-state point or a limit cycle (appearing as the total synchronization or seizure). Linking several units permits the design of a system consisting of multiple states (Koppert et al., 2014). Notably, such a system can engross a diversity of alternative oscillatory excited states, while state transitions occur solely as a consequence of external disturbances. A computational model (Bauer et al., 2017) has indicated that the addition of a global expression to the dynamics of the multistate system prevents hypersynchronous activity and discloses multiple phenomena described by the model. For example, when fitting state duration distributions to an exponential distribution, the distribution of times spent in one state will follow a particular case of the gamma distribution with less than one shape parameter. Thus, external stochastic perturbations cause transitions from one state to another. A distributed model built from complex bistable units can practically simulate the seizure onset, maintenance and termination processes (Bauer et al., 2017).

### Multistate Models—State Holding Close to the Transition Point

The bistable model formulates a valid hypothesis to assess the proximity to ictal transition even at the level of single neurons. When the system is disturbed, the closer it comes to the region splitting the normal steady-state from the oscillatory limit cycle (the model seizure), the longer is the time for responses or the time needed to return to the baseline state (Petkov et al., 2018). This result is caused by the fact that the separatrix (i.e., the boundary separating two modes of behavior in a differential equation) is diverse under an unstable asymptotic state acting as a limit cycle. Thus, the forces necessary to shift the system out of it are minute in the local network. This feature was used to develop a biomarker that can be combined with transcranial electrical stimulation (TES) or transcranial magnetic stimulation (TMS) for diagnostic and therapeutic prognosis protocols.

## Phase Detection, Phase Prediction and Time and Space Targeting

### Phase Detection and Prediction Algorithms

The phase of brain oscillations is an essential feature of neural processing (Thut et al., 2012; Maris et al., 2016). Therefore, it can act as an index of brain excitability, temporally guiding the delivery of brain stimulation. Several different algorithms have been developed to detect and predict the phase of various EEG oscillations for TES and TMS based closed-loop stimulation, as follows.

#### Fast Fourier Transform Prediction

The crucial feature of this algorithm is to use the frequency domain of the EEG signal for forwarding prediction (Mansouri et al., 2017). One specific implementation uses Laplacian montage with a central electrode of interest and eight surrounding electrodes as the brain signal for the region of interest (Shirinpour et al., 2020). The signal’s phase in the dominant frequency is estimated from the angular factor of the complex Fast Fourier Transform (FFT) signal. A sine wave of the dominant oscillation with a given frequency and phase is calculated in the earlier steps and used for forwarding prediction.

#### Auto Regressive Prediction

In this approach, the signal is predicted in the time domain (Chen et al., 2013; Zrenner et al., 2018) in the following steps. First, the Laplacian of the electrodes corresponding to the region of interest is calculated. Next, the signal is zero-phase band-pass filtered in the frequency band of interest using an finite impulse response (FIR) filter [the FIR filter is a non-recursive filter in that the output from the filter is calculated by using the current and previous inputs (Mokhatab and Poe, 2012)] and the edges of the signal are curtailed to remove edge artifacts due to filtering. The residual signal is used to calculate the coefficients for the autoregressive model [i.e., the Yule-Walker method (Walker, 1931; Yule, 2012)]. The signal is heuristically forward predicted depending upon the parameters of the Auto Regressive (AR) coefficients. The instantaneous phase of the predicted signal is calculated using the Hilbert transformation.

#### Educated Temporal Prediction

This method integrates a short training step for the algorithm before the real-time application aiming to learn the individual statistical characteristics of the oscillation of interest. It uses a robust and straightforward method to extract inter-peak intervals and their central moment (Shirinpour et al., 2020). Presuming that brain oscillations are quasi-stable over the brief measurement epochs; one can determine the characteristic interval period between subsequent signal peaks (relating to 360° in signal phase). To predict the time-point at which the next target phase, i.e., the peak, will arise, one can add the average measured period between signal peaks to the time of the last peak recorded in order to predict the next peak.

### Time-and Space-Targeting

Pathological oscillations can be modulated using open- or closed-loop approaches depending on how the stimulation is performed in the temporal domain. Analyzing various parameters of the outputs of the neuronal networks can be utilized to optimize the effect of stimulation. The feedback input allows the modulation to be time-targeted using on-demand stimulation (Berényi et al., 2012; Takeuchi and Berényi, 2020).

### Closed-Loop Interventions

Closed-loop techniques for oscillotherapeutics are brain stimulation protocols based on intrinsic biosignal feedback [e.g., EEG, electrocardiogram (ECG), LFP]. The feedback input enables on-demand targeted intervention in the temporal domain preventing over-stimulation and undesired out-of-phase interferences. Closed-loop intervention can lower the side effects of relatively intensive stimulation; in contrast, chronic stimulation using a non-responsive, open-loop method can become involuntarily excessive. Indeed, inadequate stimulation can develop adverse effects by disturbing physiological activity in the brain. Remarkably, patients advised to turn on deep brain stimulation (DBS) in an on-demand manner for essential tremor show improved long term effects compared to open-loop continuous stimulation (Kronenbuerger et al., 2006).

**FIGURE 1:**
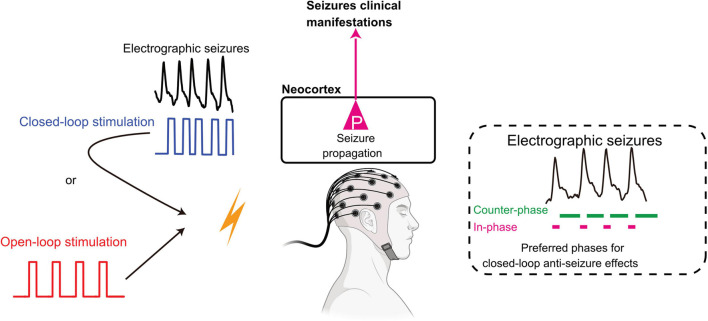
Open-loop and closed-loop interventions in epileptic seizures. Open-loop intervention delivers preset stimulation naive to the ongoing rhythmicity of brain activity., while closed-loop intervention governs stimulation pattern by the real-time processing of network oscillations. Counter-phase stimulation cancels intrinsic oscillations by destructive interference. In contrast, in-phase stimulation can enhance or restore decreased oscillations by constructive interference. See details in the main text. Adapted from Huang et al. (2019) and Takeuchi et al. (2021)

The closed-loop method can be implemented in various ways according to the characteristics and impact of the intrinsic biosignal (Figure 1). The first possible configuration is “closed-loop responsive” stimulation, whereby predefined stimulus pulses are delivered only when stimulation is required (on-demand). In this setup, biosignals are continuously monitored for the automated launch of a preset stimulation pattern. The second closed-loop configuration for brain stimulation is the “closed-loop adaptive” stimulation, where various parameters of the input biosignal gate output variables for stimulation. For example, the power of beta oscillations recorded in the subthalamic nucleus (STN) specifies the intensity of DBS in the STN for Parkinson’s disease patients (Bouthour et al., 2019). The third, most advanced implementation of closed-loop stimulation is “phase-targeting” stimulation. Conceptually, phase-targeting electrical stimulation is highly effective in suppressing pathological oscillations. In the restoration of reduced physiological oscillations, counter-phase stimulation suppresses pathological oscillations and in-phase stimulation recovers decreased physiological oscillations (Figure 1). Practically, appropriately timed phase-targeting stimulus delivery has been demonstrated to be essential for the closed-loop intervention by suppressing ongoing pathological oscillations in epilepsy that effectively shortens the duration of absence seizures in rats (Berényi et al., 2012), and can remain effective for months when used in a closed-loop manner (Kozák and Berényi, 2017). We also showed that accurate stimulus timing controlled by internal seizure dynamics is critical for the termination of epileptic seizures when applying closed-loop stimulation to the medial septum (Takeuchi et al., 2021).

### Focused Transcranial Electrical Stimulation Technologies

Transcranial electrical stimulation is a non-invasive brain stimulation protocol: as stimulation electrodes are located outside the skull, it is a low-risk and reversible adjunctive therapy. The focality of TES is poorer than DBS because of its transcranial nature. On the other hand, its diffuse modulation over the cortex may be considered as an advantage for intervention with generalized pathological oscillations hijacking wide cortical areas, as in the case of absence seizures (Berényi et al., 2012; Kozák and Berényi, 2017).

#### High-Definition Transcranial Direct Current Stimulation

Transcranial direct current stimulation (tDCS) is utilized to induce plastic changes by introducing sub-threshold membrane potential alterations in neurons of the cerebral cortex. Classical tDCS applies two large electrodes generating subthreshold depolarization of cortical neurons under the anodal electrode and hyperpolarization under the cathodal electrode, respectively. To increase the focality of tDCS, reducing the size of the large stimulus electrode placed over the target area, increasing the size of the return electrode, or changing the location of the return electrode (for example, over the arms, neck, shoulders, or knees) can be considered. An electrode configuration with improved stimulation focality has been developed based on modeling electrical field strength, termed high-definition tDCS (HD-tDCS) (Nitsche et al., 2015). Considering that the spacing between the HD-tDCS electrodes is relatively small, shunting is enhanced relative to the more conventional electrode configurations. Hence, current density has to be relatively high to generate electric fields comparable to those generated by large electrode pads with larger spacing. Studies have revealed that HD-tDCS treatment can alleviate epilepsy and pain perception (Castillo-Saavedra et al., 2016; Meiron et al., 2019).

#### High-Definition Transcranial Alternating Current Stimulation

Transcranial alternating current stimulation (tACS) is a stimulation technique that non-invasively modulates cortical activity and excitability. tACS is supposed to affect neuronal membrane potentials by oscillatory electrical stimulation using a well-defined stimulation frequency (Nitsche et al., 2015). As HD-tDCS, tACS focality can also be drastically increased by applying one stimulating electrode on the target area surrounded by multiple anti-phase returning electrodes (named as HD-tACS). Numerous cortical regions can be individually stimulated with well-defined oscillatory stimulus waveforms. This technique has been used to synchronize and desynchronize the activity of the human medial frontal cortex and the lateral PFC in the theta (∼6 Hz) frequency band resulting in the effective modulation of executive functions (Reinhart, 2017).

### Temporal Interference Stimulation

Temporal interference (TI) stimulation is a novel TES method that promises to empower DBS without affecting superficial, off-target structures (Grossman et al., 2017). TI stimulation exploits the temporal interference among two electrical fields with alternating vectorial directions using similar, but slightly different frequencies in the kHz frequency band (i.e., 2 and 2.1 kHz). During TI stimulation one delivers the brain multiple electric fields at frequencies too high to recruit neural firing, but which differ by a frequency amenable to recruit neural activity. Effective electrical stimulation of neurons is suggested to occur across a local area where the interference among the multiple fields generates an emergent electric field envelope modulated at the difference frequency (i.e., 0.1 kHz) without excessive side effects. Spatial targeting of TI is confirmed in computational models, slice experiments and in anesthetized rodents (Grossman et al., 2017; Esmaeilpour et al., 2021), spatial resolution depends on the number and alignment of electrodes over the scalp. The possible off-target effects of high-frequency electrical fields over large brain areas could present an issue as strong kHz-frequency electrical fields can block the spreading of compound action potentials in peripheral nerves (Kilgore and Bhadra, 2014). The long-term effects of kHz stimulation of TI are yet unknown. The temporal resolution of TI simulation is limited, as the generation of kHz electrical fields in short ramp-up times induces rapid, spatially unfocused activation of neurons, while slow ramp-up does not (Grossman et al., 2017). Due to this limitation, precisely timed closed-loop TI (i.e., phase-targeting stimulation) is not achievable. Accordingly, TI stimulation appears to be preferable to applications for inducing plasticity in subcortical brain regions (Chaieb et al., 2011).

#### Intersectional Short Pulse Stimulation

We have previously developed a novel TES approach (Intersectional-Short Pulse (ISP) stimulation) which allows to programmatically steer the effect of TES in the intracranial space and allows considerably higher electrical currents to be used, while preserving the high temporal precision of the stimulation (Vöröslakos et al., 2018). ISP applies a repeated sequence of brief, amplitude modulated electrical pulses through multiple independent electrode pairs. The ISP method exploits the temporal integration of the subthreshold changes induced by the multiple consecutive electrical gradients due to the capacitive properties of the neuronal membranes. Accordingly, due to this neuronal “blurring” ISP stimulation can transcranially mimic the neuronal readout that would be caused by a strong (>1 mV/mm), continuous electrical field in a target brain region to directly induce or inhibit action potentials without generating excessive current densities on the scalp (i.e., causing less peripheral effects). Using ISP, the activity of hippocampal neurons can be modulated in a hemisphere-specific way (Vöröslakos et al., 2018). In addition, the 1 Hz ISP stimulation can modulate the amplitude of alpha-band oscillations in EEG recordings of healthy volunteers in a hemisphere-and phase-specific manner (Vöröslakos et al., 2018).

The advantages of ISP compared to other electrical stimulation techniques are several. It has better spatial steerability and can be implemented with a phase-targeted closed-loop configuration with millisecond precision. Currents as much as 16 mA can be applied (one order of magnitude larger than conventional TES), but the current density on each electrode stays similar to those used by traditional TES. Identical effects (i.e., excitation or inhibition) can be simultaneously achieved on both hemispheres by appropriate electrode alignment in contrast to conventional TES, which generates opposing anodal-cathodal effects over the two hemispheres. The direction of electrical fields along the axo-dendritic axis of neurons determines whether the electrical fields activate or inhibit the target neurons (Chan and Nicholson, 1986; Liu et al., 2018). Furthermore, several distinct stimulus waveforms can be employed in an interwoven fashion, yet independently. This stimulation technique is expected to allow non-invasive on-demand closed-loop control with space-and time-targeted brain stimulation for the treatment of various neuropsychiatric disorders.

### The Matter of Stimulus Intensity/Effect Size to Reach Reliable Control on Oscillatory Network Patterns

TES applied at ± 1 mA peak intensity induces < 0.5 V/m electric fields in the human brain (Opitz et al., 2016; Huang et al., 2017; Chhatbar et al., 2018). This is enough to induce 0.1–0.2 mV alterations in the membrane potential of neurons within the stimulated area. As these alterations are markedly lower than the ∼20 mV depolarization necessary to push a neuron from its resting potential to spike threshold *in vitro*, TES is unable to obtain prompt, highly reproducible changes in spiking activity. In contrast, the mild electrical fields generated may be more efficient when applied to distract or reinforce ongoing rhythms rather than introducing novel activity patterns. Targeting the stimulation to the optimal phase of endogenous rhythms in a closed-loop implementation may be the most effective solution (Berényi et al., 2012). However, responsive implementations require in depth characterization of the altered network with simultaneous monitoring and adjustment of the relevant rhythms (Krook-Magnuson et al., 2015). This might be achieved by using mild electric fields, but other applications may require higher field intensities for improved efficacy. For example, immediate control of spiking activity (e.g., to terminate a seizure) might require field strengths larger than 5 V/m (Kozák and Berényi, 2017).

Taking into account the intrinsic circuit structures of a given brain area is an essential factor for the effective control of oscillatory networks as stimulation of structural hubs effectively modulates the ongoing oscillatory activity with high spatial propagation. The applied stimulation frequency needs to match the frequency range of activity of various elements of the targeted neuronal circuit. Supposing the targeted brain region has divergent projections to multiple brain regions, oscillatory activity applied to the target region can extend to various destination brain regions. TES of the prefrontal cortex (PFC) for the treatment of depression readily utilizes this concept because the PFC has widespread synaptic connections to limbic areas (Loo et al., 2012; Ferenczi et al., 2016). The interference of a “congestion” relay station in the brain effectively intervenes in disseminating massive oscillatory activity like epileptic seizures (Takeuchi et al., 2021). In this respect, the entorhinal cortex and the subiculum are chokepoint-like structures broadcasting the activity from the hippocampus to the neocortex. Stimulation on these structures can effectively suppress the secondary generalization of seizures originating in the hippocampus (Lu et al., 2016; Wang et al., 2017). On the other hand, the STN and the thalamus are chokepoints in post-stroke epilepsy and absence epilepsy (Paz and Huguenard, 2015).

## Future Directions

### Seizure Detection and Prediction

The real-time prediction of seizures is more challenging than detecting seizures because of atypical feature changes and smaller signal-to-noise ratios. However, the prediction would be more beneficial than detection as it enables prevention.

Attempts to develop reliable seizure prediction algorithms have an extensive history, dating back to the 1970s (Viglione and Walsh, 1975) with minimal data sets looking only at pre-seizure (preictal) events minutes to seconds before seizures. Massively evolving over the past 50 years, current methods use mathematical tools to analyze continuous days of multiscale EEG recordings (Lehnertz and Litt, 2005). One of the most salient features of seizures is their unpredictability. From a more comprehensive view, seizure prediction research has also transformed how we understand epilepsy and the basic mechanisms underlying seizure generation. Seizures were formerly considered isolated and abrupt events, but we now consider them processes that develop over space and time in epileptic networks (Burns et al., 2014). Therefore, what started as predicting seizures for clinical applications has evolved into a field committed to understanding seizure generation.

### Seizure Prediction Algorithms

Seizure prediction algorithms typically follow the same route/logic: biosignals are recorded and pre-processed, prediction features and/or pre-ictal biomarkers are then extracted. The decision system processes the temporal stream of feature prediction values and detects changes that indicate an upcoming seizure. To reach a decision, thresholds can be set for various features, or machine learning classifiers can be used to make decisions based on multiple features. The decision system then involves the advisory system, which warns the patient if a seizure is likely to occur soon. Constantly acquired biosignals, most frequently EEG or intracranial EEG, are analyzed with advanced time series analysis methods to identify predictive features. A pre-ictal biomarker is a predictive feature derived from physiological signals (for example the EEG) that becomes apparent during a defined period before a seizure but not at other times. Such a feature might or might not be visually evident, reflects underlying signals’ alterations and predicts seizures within an explicit range of values. Features are commonly used instead of the raw signals because they simplify the essential changes of the signals. A pre-ictal feature can be considered clinically beneficial as a warning system if it can be detected early enough and can minimize the time under false warning. Features evaluated for their predictive value, particularly those of EEG signals, range from simple to complex and rely on univariate, bivariate or multivariate linear, or non-linear analysis. The effectiveness of individual features for seizure prediction can be evaluated individually, but combinations of features are often delivered as inputs to machine learning algorithms, acting as pattern recognition systems. These algorithms allow the estimation of the seizure prediction properties of features in combination (Freestone et al., 2015; Brinkmann et al., 2016; Gadhoumi et al., 2016). In turn, these temporal features are utilized in decision algorithms to trigger the delivery of pharmacological or non-pharmacological control of seizures in a closed-loop system. Algorithms need additional development based on neurophysiology, multimodal imaging, seizure mechanisms, control theory and computational modeling (Kuhlmann et al., 2015). Numerous guidelines and approaches are used to develop seizure prediction algorithms (Mormann et al., 2007). These methods require receiver operating characteristic (ROC) curves that measure the true positive rate against the false-positive rate during pre-ictal or inter-ictal periods. Overall algorithm performance can be quantified and ranked by the area under the curve (AUC) for true positive vs. false-positive rates.

Biomarkers of epilepsy incorporate interictal epileptiform discharges and bursts, interictal spikes and high-frequency oscillations, which are nowadays used in diagnosis, surgical planning and treatment bearing obvious clinical significance (Matsumoto and Marsan, 1964; Schulze-Bonhage, 2016). The hope for seizure prediction was high in the early twenty first century following the development of a plethora of seizure prediction algorithms. Still, the result of stringent testing on the reliability of seizure prediction indicates no evidence of above-chance prediction (Mormann et al., 2007). No predictive feature or pre-ictal characteristic that is generic among people with epilepsy and that can predict the precise time of an individual’s subsequent seizure has been yet identified (Kuhlmann et al., 2010, 2018; Gadhoumi et al., 2015; Karoly et al., 2017; Kiral-Kornek et al., 2018; Truong et al., 2018). Thus, it is important to decide whether promising seizure predictors forecast seizures rather than detect random fluctuations in EEG signals unrelated to seizures. This principle challenge in seizure prediction requires a standardized stringent mathematical calculation of predictive performance (Mormann et al., 2007) because seizure events are sparse and interictal periods generally long. A first step for such analysis is to compare the performance of a prediction algorithm with that of a random predictor (Schelter et al., 2006; Snyder et al., 2008) that generates predictions at random times at the same rate as that of the algorithm. Where appropriate, a random predictor can be adapted to account for a subject’s diurnal variability in seizure distribution or features (Karoly et al., 2017). More evolved methods utilize Monte Carlo simulations to generate predictor substitutes, such as randomizing seizure times to generate false seizure times (Andrzejak et al., 2003, 2009; Kreuz et al., 2004). The performance of the prediction algorithm is then mathematically compared with the efficacy of these predictor substitutes. Comparing the performance of a prediction algorithm with a random predictor is algorithmically the most effective form of mathematical calculation. Substitute-based methods have higher temporal complexity but improve confidence in concluding whether an algorithm performs better than chance and can report the non-random occurrence of seizures. The importance of rigorous mathematical testing of seizure prediction algorithms is crucial for understanding the significance of the results of seizure prediction.

## Precise Localization and Targeting of a Seizure Focus in the Brain

Increasing the number of pairs of stimulating electrodes is essential for improving the spatial resolution of ISP stimulation (this is also the case for TI stimulation). A dedicated EEG cap with multiple recording and stimulating electrodes is needed for transcranial closed-loop intervention with ISP stimulation. Sub-scalp or intracranial implantation of the stimulus electrodes will boost the efficiency and focality at the expense of a more invasive intervention. The placement of stimulating electrodes must be adapted for each patient’s requirements, especially in the case of focal seizures. The target brain region should be determined by a combination of high-density EEG, functional tomography and long-term video monitoring of seizures. Structural brain imaging [i.e., magnetic resonance imaging (MRI)] is also required for planning the ISP stimulation targets. A recent study described a multi-electrode model for electrical stimulation (Huang et al., 2019). Mathematical investigations (solving linear programming problems) showed a patient-specific MRI-based model to determine the electrode positions and current intensities that optimize the induced electric fields in either intensity or focality at the target location. In addition, the achievable focality is limited by the safety constraint on maximum currents (Dmochowski et al., 2011). Although electrical artifacts of ISP stimulation are smaller than those of conventional TES, feedforward removal of gross artifacts from applied currents is required (Vöröslakos et al., 2018; Kohli and Casson, 2019). Optimizing stimulation parameters (duration, intensity, etc.) is crucial for optimal performance. Empirical optimization presently used by clinicians is a labor and time-consuming process. Machine learning algorithms could be utilized instead for optimizing closed-loop ISP stimulation (timing and parameters) for the control of epileptic seizures.

## Closed-Loop Implementations

The first studies using early seizure-detection algorithms in combination with responsive brain stimulation have yielded positive results (Kossoff et al., 2004; Fountas et al., 2005; Osorio et al., 2005). For any responsive brain-stimulation configuration, a key issue is the placement of both afferent and efferent electrodes, that is, electrodes for detecting a pre-seizure state and stimulation electrodes, respectively. The location and number of electrodes used may be essential for the early detection of an impending seizure followed by locally applied, spatially constrained stimulation, in a way that the patient does not wittingly perceive the intervention.

The ultimate aim in designing a reliable seizure-prediction algorithm can be seen in a device capable of warning of an impending seizure and preventing it from happening. An ideal intervention system would control the development of an episode before the onset of the clinical symptoms. Its tolerance toward false alarms leading to unnecessary interventions would depend on the magnitude of side effects. The principal feasibility of different seizure-intervention strategies such as local application of short-acting drugs (Stein et al., 2000), electrical stimulation techniques (Berényi et al., 2012), local cooling (Hill et al., 2000), or biofeedback operant conditioning (Sterman, 2000) has been described in the literature. Presently, much research is directed toward designing a closed-loop intervention system using deep brain or transcranial stimulation (Morrell, 2006; Kozák and Berényi, 2017; Takeuchi et al., 2021). Such an EEG-based closed-loop stimulation system could be based either on prediction algorithms or algorithms for early seizure detection. Nowadays, prediction algorithms are limited in performance to verify clinical trials with closed-loop stimulation using the techniques described above. For early seizure-detection algorithms, the challenge is whether an intervention after the onset of an electrographic seizure can prevent its full clinical manifestation or whether the brain has already passed the “point of no return.” Detection algorithms should be optimized to be implemented into a micro processing unit. Extensive parallelization will be necessary to enable real-time computation in a small device with a limited clock rate without substantial delays. Phase-locked stimulation is essential for efficient intervention with pathological oscillations. New algorithms for instantaneous phase calculation will be valuable if implemented in the closed-loop system for efficient intervention in pathological oscillations (Mansouri et al., 2017). Even if appropriate offline modeling methods are time demanding, the online detection of specific oscillatory patterns based on the constituted model can be achievable as it does not demand calculations as intense as the modeling process itself. A reduction in the dimensionality and complexity of the model may be required for online intervention.

## Author Contributions

TF and AB developed the idea. TF prepared the figure. TF and ML wrote the original draft. TF, ML, and AB discussed and commented on the manuscript. All authors contributed to the article and approved the submitted version.

## Conflict of Interest

AB was the owner of Amplipex Llc. and a shareholder of Neunos Ltd., Szeged, Hungary, manufacturers of signal-multiplexed neuronal amplifiers and neurostimulator devices. The remaining authors declare that the research was conducted in the absence of any commercial or financial relationships that could be construed as a potential conflict of interest.

## Publisher’s Note

All claims expressed in this article are solely those of the authors and do not necessarily represent those of their affiliated organizations, or those of the publisher, the editors and the reviewers. Any product that may be evaluated in this article, or claim that may be made by its manufacturer, is not guaranteed or endorsed by the publisher.

## Abbreviations

AP: action potential
AR: autoregressive
AUC: area under the curve
CRG: Cognitive Rhythm Generator
DBS: deep brain stimulation
DLPFC: dorsolateral prefrontal cortex
ECG: electrocardiogram
EEG: electroencephalogram
ETP: educated temporal prediction
EPSC: excitatory postsynaptic current
FC: functional connectivity
FFT: Fast Fourier transform
ISP: intersectional-short pulse
LFP: local field potential
HD-tDCS: high definition transcranial direct current stimulation
HD-tACS: high definition transcranial alternating current stimulation
MDD: major depressive disorder
MRI: magnetic resonance imaging
PFC: prefrontal cortex
ROC: receiver operating characteristic
SED: spontaneous epileptiform discharge
STN: subthalamic nucleus
tACS: transcranial alternating current stimulation
tDCS: transcranial direct current stimulation
TES: transcranial electrical stimulation
TI: temporal interference
TLE: temporal lobe epilepsy
TMS: transcranial magnetic stimulation.

## References

[B1] American Psychiatric Association (2013). *Diagnostic and Statistical Manual of Mental Disorders*, 5th Edn. Arlington, VA: American Psychiatric Association.

[B2] Amzica F. Steriade M. (2000). Neuronal and glial membrane potentials during sleep and paroxysmal oscillations in the neocortex. *J.Neurosci.* 20 6648–6665. 10.1523/jneurosci.20-17-06648.2000 10964970 PMC6772987

[B3] Anastassiou C. A. Perin R. Markram H. Koch C. (2011). Ephaptic coupling of cortical neurons. *Nat. Neurosci.* 14 217–224. 10.1038/nn.2727 21240273

[B4] Andrzejak R. G. Chicharro D. Elger C. E. Mormann F. (2009). Seizure prediction: any better than chance? *Clin. Neurophysiol.* 120 1465–1478. 10.1016/j.clinph.2009.05.019 19576849

[B5] Andrzejak R. G. Mormann F. Kreuz T. Rieke C. Kraskov A. Elger C. E. (2003). Testing the null hypothesis of the nonexistence of a preseizure state. *Phy. Rev. E* 67:4. 10.1103/PhysRevE.67.010901 12636484

[B6] Avoli M. Louvel J. Pumain R. Köhling R. (2005). Cellular and molecular mechanisms of epilepsy in the human brain. *Prog. Neurobiol.* 77 166–200. 10.1016/j.pneurobio.2005.09.006 16307840

[B7] Baskaran A. Milev R. McIntyre R. S. (2012). The neurobiology of the EEG biomarker as a predictor of treatment response in depression. *Neuropharmacology* 63 507–513. 10.1016/j.neuropharm.2012.04.021 22569197

[B8] Bauer P. R. Thijs R. D. Lamberts R. J. Velis D. N. Visser G. H. Tolner E. A. (2017). Dynamics of convulsive seizure termination and postictal generalized EEG suppression. *Brain* 140 655–668. 10.1093/brain/aww322 28073789 PMC5837448

[B9] Bekenstein J. W. Lothman E. W. (1993). Dormancy of inhibitory interneurons in a model of temporal lobe epilepsy. *Science* 259 97–100. 10.1126/science.8093417 8093417

[B10] Berényi A. Belluscio M. Mao D. Buzsáki G. (2012). Closed-loop control of epilepsy by transcranial electrical stimulation. *Science* 337 735–737. 10.1126/science.1223154 22879515 PMC4908579

[B11] Berg A. T. Vickrey B. G. Langfitt J. T. Sperling M. R. Walczak T. S. Shinnar S. (2003). The multicenter study of epilepsy surgery: recruitment and selection for surgery. *Epilepsia* 44 1425–1433. 10.1046/j.1528-1157.2003.24203.x 14636351

[B12] Bettus G. Bartolomei F. Confort-Gouny S. Guedj E. Chauvel P. Cozzone P. J. (2010). Role of resting state functional connectivity MRI in presurgical investigation of mesial temporal lobe epilepsy. *J. Neurol. Neurosurg. Psychiatry* 81 1147–1154. 10.1136/jnnp.2009.191460 20547611

[B13] Bezaire M. J. Raikov I. Burk K. Vyas D. Soltesz I. (2016). Interneuronal mechanisms of hippocampal theta oscillations in a full-scale model of the rodent CA1 circuit. *Elife* 5:e18566. 10.7554/eLife.18566 28009257 PMC5313080

[B14] Bone B. Fogarasi A. Schulz R. Gyimesi C. Kalmar Z. Kovacs N. (2012). Secondarily generalized seizures in temporal lobe epilepsy. *Epilepsia* 53 817–824. 10.1111/j.1528-1167.2012.03435.x 22429112

[B15] Bouthour W. Mégevand P. Donoghue J. Lüscher C. Birbaumer N. Krack P. (2019). Biomarkers for closed-loop deep brain stimulation in Parkinson disease and beyond. *Nat. Rev. Neurol.* 15 343–352. 10.1038/s41582-019-0166-4 30936569

[B16] Bower M. R. Stead M. Meyer F. B. Marsh W. R. Worrell G. A. (2012). Spatiotemporal neuronal correlates of seizure generation in focal epilepsy. *Epilepsia* 53 807–816. 10.1111/j.1528-1167.2012.03417.x 22352423 PMC3339564

[B17] Brinkmann B. H. Wagenaar J. Abbot D. Adkins P. Bosshard S. C. Chen M. (2016). Crowdsourcing reproducible seizure forecasting in human and canine epilepsy. *Brain* 139 1713–1722. 10.1093/brain/aww045 27034258 PMC5022671

[B18] Burns S. P. Santaniello S. Yaffe R. B. Jouny C. C. Crone N. E. Bergey G. K. (2014). Network dynamics of the brain and influence of the epileptic seizure onset zone. *Proc. Natl. Acad. Sci. U.S.A.* 111 E5321–E5330. 10.1073/pnas.1401752111 25404339 PMC4267355

[B19] Buzsáki G. (2009). *Rhythms of the Brain.* Oxford: Oxford University Press.

[B20] Buzsáki G. Anastassiou C. A. Koch C. (2012). The origin of extracellular fields and currents-EEG, ECoG, LFP and spikes. *Nat. Rev. Neurosci.* 13 407–420. 10.1038/nrn3241 22595786 PMC4907333

[B21] Buzsáki G. Draguhn A. (2004). Neuronal olscillations in cortical networks. *Science* 304 1926–1929. 10.1126/science.1099745 15218136

[B22] Buzsáki G. Watson B. O. (2012). Brain rhythms and neural syntax: implications for efficient coding of cognitive content and neuropsychiatric disease. *Dialogues Clin. Neurosci.* 14 345–367. 10.31887/dcns.2012.14.4/gbuzsaki 23393413 PMC3553572

[B23] Carmignoto G. Haydon P. G. (2012). Astrocyte calcium signaling and epilepsy. *Glia* 60 1227–1233. 10.1002/glia.22318 22389222 PMC4532388

[B24] Castillo-Saavedra L. Gebodh N. Bikson M. Diaz-Cruz C. Brandao R. Coutinho L. (2016). Clinically effective treatment of fibromyalgia pain with high-definition transcranial direct current stimulation: phase II open-label dose optimization. *J. Pain* 17 14–26. 10.1016/j.jpain.2015.09.009 26456677 PMC5777157

[B25] Chaieb L. Antal A. Paulus W. (2011). Transcranial alternating current stimulation in the low kHz range increases motor cortex excitability. *Restor. Neurol. Neurosci.* 29 167–175. 10.3233/RNN-2011-0589 21586823

[B26] Chan C. Y. Nicholson C. (1986). Modulation by applied electric fields of Purkinje and stellate cell activity in the isolated turtle cerebellum. *J. Physiol.* 371 89–114. 10.1113/jphysiol.1986.sp015963 3701658 PMC1192712

[B27] Chen L. L. Madhavan R. Rapoport B. I. Anderson W. S. (2013). Real-time brain oscillation detection and phase-locked stimulation using autoregressive spectral estimation and time-series forward prediction. *IEEE Trans. Biomed. Eng.* 60 753–762. 10.1109/TBME.2011.2109715 21292589 PMC3371105

[B28] Chhatbar P. Y. Kautz S. A. Takacs I. Rowland N. C. Revuelta G. J. George M. S. (2018). Evidence of transcranial direct current stimulation-generated electric fields at subthalamic level in human brain in vivo. *Brain Stimul.* 11 727–733. 10.1016/j.brs.2018.03.006 29576498 PMC6019625

[B29] Cisler J. M. Sigel B. A. Kramer T. L. Smitherman S. Vanderzee K. Pemberton J. (2015). Amygdala response predicts trajectory of symptom reduction during trauma-focused cognitive-behavioral therapy among adolescent girls with PTSD. *J. Psychiatr. Res.* 71 33–40. 10.1016/j.jpsychires.2015.09.011 26522869 PMC4826076

[B30] Clancy K. Ding M. Bernat E. Schmidt N. B. Li W. (2017). Restless “rest”: Intrinsic sensory hyperactivity and disinhibition in post-traumatic stress disorder. *Brain* 140 2041–2050. 10.1093/brain/awx116 28582479 PMC6059177

[B31] Cooper R. A. Ritchey M. (2019). Cortico-hippocampal network connections support the multidimensional quality of episodic memory. *Elife* 8:e45591. 10.7554/eLife.45591 30900990 PMC6450667

[B32] Derchansky M. Rokni D. Rick J. T. Wennberg R. Bardakjian B. L. Zhang L. (2006). Bidirectional multisite seizure propagation in the intact isolated hippocampus: the multifocality of the seizure “focus.”. *Neurobiol. Dis.* 23 312–328. 10.1016/j.nbd.2006.03.014 16815026

[B33] Derecki N. C. Cronk J. C. Lu Z. Xu E. Abbott S. B. G. Guyenet P. G. (2012). Wild-type microglia arrest pathology in a mouse model of Rett syndrome. *Nature* 484 105–109. 10.1038/nature10907 22425995 PMC3321067

[B34] Dmochowski J. P. Datta A. Bikson M. Su Y. Parra L. C. (2011). Optimized multi-electrode stimulation increases focality and intensity at target. *J. Neural Eng.* 8:046011. 10.1088/1741-2560/8/4/046011 21659696

[B35] Duckrow R. B. Spencer S. S. (1992). Regional coherence and the transfer of ictal activity during seizure onset in the medial temporal lobe. *Electroencephalogr. Clin. Neurophysiol.* 82 415–422. 10.1016/0013-4694(92)90046-K 1375548

[B36] Eidelman-Rothman M. Levy J. Feldman R. (2016). Alpha oscillations and their impairment in affective and post-traumatic stress disorders. *Neurosci. Biobehav. Rev.* 68 794–815. 10.1016/j.neubiorev.2016.07.005 27435239

[B37] Engel A. K. Fries P. Singer W. (2001). Dynamic predictions: oscillations and synchrony in top–down processing. *Nat. Rev. Neurosci.* 2 704–716. 10.1038/35094565 11584308

[B38] Englot D. J. Konrad P. E. Morgan V. L. (2016). Regional and global connectivity disturbances in focal epilepsy, related neurocognitive sequelae, and potential mechanistic underpinnings. *Epilepsia* 57 1546–1557. 10.1111/epi.13510 27554793 PMC5056148

[B39] Esmaeilpour Z. Kronberg G. Reato D. Parra L. C. Bikson M. (2021). Temporal interference stimulation targets deep brain regions by modulating neural oscillations. *Brain Stimul.* 14 55–65. 10.1016/j.brs.2020.11.007 33186778 PMC9382891

[B40] Eyo U. B. Peng J. Swiatkowski P. Mukherjee A. Bispo A. Wu L. J. (2014). Neuronal hyperactivity recruits microglial processes via neuronal NMDA receptors and microglial P2Y12 receptors after status epilepticus. *J. Neurosci.* 34 10528–10540. 10.1523/JNEUROSCI.0416-14.2014 25100587 PMC4200107

[B41] Farah F. H. Grigorovsky V. Bardakjian B. L. (2019). Coupled oscillators model of hyperexcitable neuroglial networks. *Int. J.Neural Syst.* 29:1850041. 10.1142/S0129065718500417 30415633

[B42] Fellin T. Pascual O. Gobbo S. Pozzan T. Haydon P. G. Carmignoto G. (2004). Neuronal synchrony mediated by astrocytic glutamate through activation of extrasynaptic NMDA receptors. *Neuron* 43 729–743. 10.1016/j.neuron.2004.08.011 15339653

[B43] Ferenczi E. A. Zalocusky K. A. Liston C. Grosenick L. Warden M. R. Amatya D. (2016). Prefrontal cortical regulation of brainwide circuit dynamics and reward-related behavior. *Science* 351:6268. 10.1126/science.aac9698 26722001 PMC4772156

[B44] Fisher R. S. Acevedo C. Arzimanoglou A. Bogacz A. Cross J. H. Elger C. E. (2014). ILAE official report: a practical clinical definition of epilepsy. *Epilepsia* 55 475–482. 10.1111/epi.12550 24730690

[B45] Fisher R. S. Van Emde Boas W. Blume W. Elger C. Genton P. Lee P. (2005). Epileptic seizures and epilepsy: definitions proposed by the International League Against Epilepsy (ILAE) and the International Bureau for Epilepsy (IBE). *Epilepsia* 46 470–472. 10.1111/j.0013-9580.2005.66104.x 15816939

[B46] Fitzgerald P. J. Watson B. O. (2018). Gamma oscillations as a biomarker for major depression: an emerging topic. *Transl. Psychiatry* 8:177. 10.1038/s41398-018-0239-y 30181587 PMC6123432

[B47] Fountas K. N. Smith J. R. Murro A. M. Politsky J. Park Y. D. Jenkins P. D. (2005). Implantation of a closed-loop stimulation in the management of medically refractory focal epilepsy: a technical note. *Stereotact. Funct. Neurosurg.* 83 153–158. 10.1159/000088656 16205108

[B48] Freestone D. R. Karoly P. J. Peterson A. D. H. Kuhlmann L. Lai A. Goodarzy F. (2015). Seizure prediction: science fiction or soon to become reality? *Curr. Neurol. Neurosci. Rep.* 15:73. 10.1007/s11910-015-0596-3 26404726

[B49] Gadhoumi K. Gotman J. Lina J. M. (2015). Scale invariance properties of intracerebral eeg improve seizure prediction in mesial temporal lobe epilepsy. *PLoS One* 10:e0121182. 10.1371/journal.pone.0121182 25867083 PMC4395084

[B50] Gadhoumi K. Lina J. M. Mormann F. Gotman J. (2016). Seizure prediction for therapeutic devices: a review. *J. Neurosci. Methods* 260 270–282. 10.1016/j.jneumeth.2015.06.010 26099549

[B51] Gast H. Niediek J. Schindler K. Boström J. Coenen V. A. Beck H. (2016). Burst firing of single neurons in the human medial temporal lobe changes before epileptic seizures. *Clin. Neurophysiol.* 127 3329–3334. 10.1016/j.clinph.2016.08.010 27592159

[B52] Glauser T. Ben-Menachem E. Bourgeois B. Cnaan A. Chadwick D. Guerreiro C. (2006). ILAE treatment guidelines: evidence-based analysis of antiepileptic drug efficacy and effectiveness as initial monotherapy for epileptic seizures and syndromes. *Epilepsia* 47 1094–1120. 10.1111/j.1528-1167.2006.00585.x 16886973

[B53] Grigorovsky V. Bardakjian B. L. (2018). Neuro-Glial network model of postictal generalized EEG suppression (PGES). *Annu. Int. Conf. IEEE Eng. Med. Biol. Soc.* 2018 2044–2047. 10.1109/EMBC.2018.8512661 30440803

[B54] Grossman N. Bono D. Dedic N. Kodandaramaiah S. B. Rudenko A. Suk H. J. (2017). Noninvasive deep brain stimulation via temporally interfering electric fields. *Cell* 169 1029–1041.e16. 10.1016/j.cell.2017.05.024 28575667 PMC5520675

[B55] Hamberger M. J. Drake E. B. (2006). Cognitive functioning following epilepsy surgery. *Curr. Neurol. Neurosci. Rep.* 6 319–326. 10.1007/s11910-006-0025-8 16822353

[B56] Hill M. W. Wong M. Amarakone A. Rothman S. M. (2000). Rapid cooling aborts seizure-like activity in rodent hippocampal-entorhinal slices. *Epilepsia* 41 1241–1248. 10.1111/j.1528-1157.2000.tb04601.x 11051118

[B57] Holmes M. Folley B. S. Sonmezturk H. H. Gore J. C. Kang H. Abou-Khalil B. (2014). Resting state functional connectivity of the hippocampus associated with neurocognitive function in left temporal lobe epilepsy. *Hum. Brain Mapp.* 35 735–744. 10.1002/hbm.22210 23124719 PMC3915042

[B58] Holmes M. D. Miles A. N. Dodrill C. B. Ojemann G. A. Wilensky A. J. (2003). Identifying potential surgical candidates in patients with evidence of bitemporal epilepsy. *Epilepsia* 44 1075–1079. 10.1046/j.1528-1157.2003.58302.x 12887439

[B59] Hong G. Lieber C. M. (2019). Novel electrode technologies for neural recordings. *Nat. Rev. Neurosci.* 20 330–345. 10.1038/s41583-019-0140-6 30833706 PMC6531316

[B60] Huang M. X. Yurgil K. A. Robb A. Angeles A. Diwakar M. Risbrough V. B. (2014). Voxel-wise resting-state MEG source magnitude imaging study reveals neurocircuitry abnormality in active-duty service members and veterans with PTSD. *Neuroimage* 5 408–419. 10.1016/j.nicl.2014.08.004 25180160 PMC4145534

[B61] Huang Y. Datta A. Bikson M. Parra L. C. (2019). Realistic volumetric-approach to simulate transcranial electric stimulation - ROAST–a fully automated open-source pipeline. *J. Neural. Eng.* 16:056006. 10.1088/1741-2552/ab208d 31071686 PMC7328433

[B62] Huang Y. Liu A. A. Lafon B. Friedman D. Dayan M. Wang X. (2017). Measurements and models of electric fields in the in vivo human brain during transcranial electric stimulation. *Elife* 6:e18834. 10.7554/eLife.18834 28169833 PMC5370189

[B63] Izhikevich E. M. (2001). Synchronization of elliptic bursters. *SIAM Rev.* 43 315–344. 10.1137/S0036144500382064

[B64] Ji K. Akgul G. Wollmuth L. P. Tsirka S. E. (2013). Microglia actively regulate the number of functional synapses. *PLoS One* 8:e56293. 10.1371/journal.pone.0056293 23393609 PMC3564799

[B65] Kalitzin S. Koppert M. Petkov G. Velis D. da Silva F. L. (2011). Computational model prospective on the observation of proictal states in epileptic neuronal systems. *Epilepsy Behav.* 22(Suppl. 1) S102–S109. 10.1016/j.yebeh.2011.08.017 22078510

[B66] Karoly P. J. Ung H. Grayden D. B. Kuhlmann L. Leyde K. Cook M. J. (2017). The circadian profile of epilepsy improves seizure forecasting. *Brain* 140 2169–2182. 10.1093/brain/awx173 28899023

[B67] Kilgore K. L. Bhadra N. (2014). Reversible nerve conduction block using kilohertz frequency alternating current. *Neuromodulation* 17 242–255. 10.1111/ner.12100 23924075 PMC3834124

[B68] Kiral-Kornek I. Roy S. Nurse E. Mashford B. Karoly P. Carroll T. (2018). Epileptic seizure prediction using big data and deep learning: toward a mobile system. *EBioMedicine* 27 103–111. 10.1016/j.ebiom.2017.11.032 29262989 PMC5828366

[B69] Koek R. J. Schwartz H. N. Scully S. Langevin J. P. Spangler S. Korotinsky A. (2016). Treatment-refractory posttraumatic stress disorder (TRPTSD): a review and framework for the future. *Prog. Neuropsychopharmacol. Biol. Psychiatry* 70 170–218. 10.1016/j.pnpbp.2016.01.015 26854815

[B70] Kohli S. Casson A. J. (2019). Removal of gross artifacts of transcranial alternating current stimulation in simultaneous EEG monitoring. *Sensors* 19:190. 10.3390/s19010190 30621077 PMC6338981

[B71] Koppert M. Kalitzin S. Velis D. Lopes Da Silva F. Viergever M. A. (2014). Dynamics of collective multi-stability in models of multi-unit neuronal systems. *Int. J. Neural. Syst.* 24:1430004. 10.1142/S0129065714300046 24475896

[B72] Kossoff E. H. Ritzl E. K. Politsky J. M. Murro A. M. Smith J. R. Duckrow R. B. (2004). Effect of an external responsive neurostimulator on seizures and electrographic discharges during subdural electrode monitoring. *Epilepsia* 45 1560–1567. 10.1111/j.0013-9580.2004.26104.x 15571514

[B73] Kozák G. Berényi A. (2017). Sustained efficacy of closed loop electrical stimulation for long-term treatment of absence epilepsy in rats. *Sci. Rep.* 7:6300. 10.1038/s41598-017-06684-0 28740261 PMC5524708

[B74] Kreuz T. Andrzejak R. G. Mormann F. Kraskov A. Stögbauer H. Elger C. E. (2004). Measure profile surrogates: a method to validate the performance of epileptic seizure prediction algorithms. *Phys. Rev. E* 69:9. 10.1103/PhysRevE.69.061915 15244625

[B75] Kronenbuerger M. Fromm C. Block F. Coenen V. A. Rohde I. Rohde V. (2006). On-demand deep brain stimulation for essential tremor: a report on four cases. *Mov. Disord.* 21 401–405. 10.1002/mds.20714 16211619

[B76] Krook-Magnuson E. Gelinas J. N. Soltesz I. Buzsáki G. (2015). Neuroelectronics and biooptics: closed-loop technologies in neurological disorders. *JAMA Neurol.* 72 823–829. 10.1001/jamaneurol.2015.0608 25961887 PMC4501886

[B77] Kuhlmann L. Freestone D. Lai A. Burkitt A. N. Fuller K. Grayden D. B. (2010). Patient-specific bivariate-synchrony-based seizure prediction for short prediction horizons. *Epilepsy Res.* 91 214–231. 10.1016/j.eplepsyres.2010.07.014 20724110

[B78] Kuhlmann L. Grayden D. B. Wendling F. Schiff S. J. (2015). Role of multiple-scale modeling of epilepsy in seizure forecasting. *J. Clin. Neurophysiol.* 32 220–226. 10.1097/WNP.0000000000000149 26035674 PMC4455036

[B79] Kuhlmann L. Karoly P. Freestone D. R. Brinkmann B. H. Temko A. Barachant A. (2018). Epilepsyecosystem.org: crowd-sourcing reproducible seizure prediction with long-term human intracranial EEG. *Brain* 141 2619–2630. 10.1093/brain/awy210 30101347 PMC6136083

[B80] Lambrecq V. Lehongre K. Adam C. Frazzini V. Mathon B. Clemenceau S. (2017). Single-unit activities during the transition to seizures in deep mesial structures. *Ann. Neurol.* 82 1022–1028. 10.1002/ana.25111 29205475

[B81] Lehnertz K. Litt B. (2005). The first international collaborative workshop on seizure prediction: summary and data description. *Clin. Neurophysiol.* 116 493–505. 10.1016/j.clinph.2004.08.020 15721063

[B82] Leuchter A. F. Hunter A. M. Krantz D. E. Cook I. A. (2015). Rhythms and blues: modulation of oscillatory synchrony and the mechanism of action of antidepressant treatments. *Ann. N. Y. Acad. Sci.* 1344 78–91. 10.1111/nyas.12742 25809789 PMC4412810

[B83] Li Y. Du X. F. Liu C. S. Wen Z. L. Du J. L. (2012). Reciprocal regulation between resting microglial dynamics and neuronal activity in vivo. *Dev. Cell* 23 1189–1202. 10.1016/j.devcel.2012.10.027 23201120

[B84] Liu A. Vöröslakos M. Kronberg G. Henin S. Krause M. R. Huang Y. (2018). Immediate neurophysiological effects of transcranial electrical stimulation. *Nat. Commun.* 9:5092. 10.1038/s41467-018-07233-7 30504921 PMC6269428

[B85] Loo C. K. Alonzo A. Martin D. Mitchell P. B. Galvez V. Sachdev P. (2012). Transcranial direct current stimulation for depression: 3-Week, randomised, sham-controlled trial. *Br. J. Psychiatry* 200 52–59. 10.1192/bjp.bp.111.097634 22215866

[B86] Lopes da Silva F. Blanes W. Kalitzin S. N. Parra J. Suffczynski P. Velis D. N. (2003). Epilepsies as dynamical diseases of brain systems: basic models of the transition between normal and epileptic activity. *Epilepsia* 44(Suppl. 12) 72–83. 10.1111/j.0013-9580.2003.12005.x 14641563

[B87] Lu Y. Zhong C. Wang L. Wei P. He W. Huang K. (2016). Optogenetic dissection of ictal propagation in the hippocampal-entorhinal cortex structures. *Nat. Commun.* 7:10962. 10.1038/ncomms10962 26997093 PMC4802168

[B88] Mansouri F. Dunlop K. Giacobbe P. Downar J. Zariffa J. (2017). A fast EEG forecasting algorithm for phase-locked transcranial electrical stimulation of the human brain. *Front. Neurosci.* 11:401. 10.3389/fnins.2017.00401 28775678 PMC5517498

[B89] Maris E. Fries P. van Ede F. (2016). Diverse phase relations among neuronal rhythms and their potential function. *Trends Neurosci.* 39 86–99. 10.1016/j.tins.2015.12.004 26778721

[B90] Massey C. A. Sowers L. P. Dlouhy B. J. Richerson G. B. (2014). Mechanisms of sudden unexpected death in epilepsy: the pathway to prevention. *Nat. Rev. Neurol.* 10 271–282. 10.1038/nrneurol.2014.64 24752120 PMC4565133

[B91] Mathalon D. H. Sohal V. S. (2015). Neural oscillations and synchrony in brain dysfunction and neuropsychiatric disorders it’s about time. *JAMA Psychiatry* 72 840–844. 10.1001/jamapsychiatry.2015.0483 26039190

[B92] Matsumoto H. Marsan C. A. (1964). Cortical cellular phenomena in experimental epilepsy: ictal manifestations. *Exp. Neurol.* 9 305–326. 10.1016/0014-4886(64)90026-3 14142796

[B93] McNaughton B. L. Barnes C. A. O’Keefe J. (1983). The contributions of position, direction, and velocity to single unit activity in the hippocampus of freely-moving rats. *Exp. Brain Res.* 52 41–49. 10.1007/BF00237147 6628596

[B94] Meindertsma H. Steenbeek H. (2012). Application of skill theory to compare scientific reasoning of young children in different tasks. *Neth. J. Psychol.* 67 9–19.

[B95] Meiron O. Gale R. Namestnic J. Bennet-Back O. Gebodh N. Esmaeilpour Z. (2019). Antiepileptic effects of a novel noninvasive neuromodulation treatment in a subject with early-onset epileptic encephalopathy: case report with 20 sessions of HDTDCS intervention. *Front. Neurosci.* 13:547. 10.3389/fnins.2019.00547 31191235 PMC6548848

[B96] Mennella R. Patron E. Palomba D. (2017). Frontal alpha asymmetry neurofeedback for the reduction of negative affect and anxiety. *Behav. Res. Ther.* 92 32–40. 10.1016/j.brat.2017.02.002 28236680

[B97] Mokhatab S. Poe W. A. (eds) (2012). “Process modeling in the natural gas processing industry,” in *Handbook of Natural Gas Transmission and Processing*, (Waltham, MA: Gulf Professional Publishing), 511–541. 10.1016/b978-0-12-386914-2.00015-7

[B98] Morgan V. L. Chang C. Englot D. J. Rogers B. P. (2020a). Temporal lobe epilepsy alters spatio-temporal dynamics of the hippocampal functional network. *Neuroimage Clin.* 26:102254. 10.1016/j.nicl.2020.102254 32251905 PMC7132094

[B99] Morgan V. L. Rogers B. P. Anderson A. W. Landman B. A. Englot D. J. (2020b). Divergent network properties that predict early surgical failure versus late recurrence in temporal lobe epilepsy. *J. Neurosurg.* 132 1324–1333. 10.3171/2019.1.JNS182875 30952126 PMC6778487

[B100] Morgan V. L. Englot D. J. Rogers B. P. Landman B. A. Cakir A. Abou-Khalil B. W. (2017). Magnetic resonance imaging connectivity for the prediction of seizure outcome in temporal lobe epilepsy. *Epilepsia* 58 1251–1260. 10.1111/epi.13762 28448683 PMC5498250

[B101] Mormann F. Andrzejak R. G. Elger C. E. Lehnertz K. (2007). Seizure prediction: the long and winding road. *Brain* 130 314–333. 10.1093/brain/awl241 17008335

[B102] Morrell M. (2006). Brain stimulation for epilepsy: can scheduled or responsive neurostimulation stop seizures? *Curr. Opin. Neurol.* 19 164–168. 10.1097/01.wco.0000218233.60217.84 16538091

[B103] Morrell M. J. (2011). Responsive cortical stimulation for the treatment of medically intractable partial epilepsy. *Neurology* 77 1295–1304. 10.1212/WNL.0b013e3182302056 21917777

[B104] Nitsche M. A. Polania R. Kuo M. F. (2015). “Transcranial direct current stimulation: modulation of brain pathways and potential clinical applications,” in *Brain Stimulation: Methodologies and Interventions*, ed. Reti I. M. (Hoboken, NJ: John Wiley & Sons, Inc.), 233–254. 10.1002/9781118568323.ch13

[B105] Opitz A. Falchier A. Yan C. G. Yeagle E. M. Linn G. S. Megevand P. (2016). Spatiotemporal structure of intracranial electric fields induced by transcranial electric stimulation in humans and nonhuman primates. *Sci. Rep.* 6:31236. 10.1038/srep31236 27535462 PMC4989141

[B106] Osorio I. Frei M. G. Sunderam S. Giftakis J. Bhavaraju N. C. Schaffner S. F. (2005). Automated seizure abatement in humans using electrical stimulation. *Ann. Neurol.* 57 258–268. 10.1002/ana.20377 15668970

[B107] Paz J. T. Huguenard J. R. (2015). Microcircuits and their interactions in epilepsy: is the focus out of focus? *Nat. Neurosci.* 18 351–359. 10.1038/nn.3950 25710837 PMC4561622

[B108] Perea G. Navarrete M. Araque A. (2009). Tripartite synapses: astrocytes process and control synaptic information. *Trends Neurosci.* 32 421–431. 10.1016/j.tins.2009.05.001 19615761

[B109] Petkov G. Kalitzin S. Demuru M. Widman G. Suffczynski P. Lopes Da Silva F. (2018). Computational model exploration of stimulation based paradigm for detection of epileptic systems. *Front. Artif. Intell. Appl.* 310:324–335. 10.3233/978-1-61499-929-4-324

[B110] Reinhart R. M. G. (2017). Disruption and rescue of interareal theta phase coupling and adaptive behavior. *Proc. Natl. Acad Sci. U.S.A.* 114 11542–11547. 10.1073/pnas.1710257114 29073084 PMC5664527

[B111] Schelter B. Winterhalder M. Maiwald T. Brandt A. Schad A. Schulze-Bonhage A. (2006). Testing statistical significance of multivariate time series analysis techniques for epileptic seizure prediction. *Chaos* 16:013108. 10.1063/1.2137623 16599739

[B112] Schulze-Bonhage A. (2016). “An introduction to epileptiform activities and seizure patterns obtained by scalp and invasive eeg recordings,” in *Epilepsy: The Intersection of Neurosciences, Biology, Mathematics, Engineering, and Physics* (Hoboken, NJ: CRC Press), 51–64. 10.1201/b10866-9

[B113] Seifert G. Carmignoto G. Steinhäuser C. (2010). Astrocyte dysfunction in epilepsy. *Brain Res. Rev.* 63 212–221. 10.1016/j.brainresrev.2009.10.004 19883685

[B114] Shirinpour S. Alekseichuk I. Mantell K. Opitz A. (2020). Experimental evaluation of methods for real-time EEG phase-specific transcranial magnetic stimulation. *J. Neural Eng.* 17 1–13. 10.1088/1741-2552/ab9dba 32554882 PMC8293904

[B115] Sik A. Penttonen M. Ylinen A. Buzsáki G. (1995). Hippocampal CA1 interneurons: an in vivo intracellular labeling study. *J. Neurosci.* 15 6651–6665. 10.1523/jneurosci.15-10-06651.1995 7472426 PMC6577981

[B116] Snyder D. E. Echauz J. Grimes D. B. Litt B. (2008). The statistics of a practical seizure warning system. *J. Neural Eng.* 5 392–401. 10.1088/1741-2560/5/4/004 18827312 PMC2888045

[B117] Staba R. J. Wilson C. L. Bragin A. Fried I. Engel J. (2002). Sleep states differentiate single neuron activity recorded from human epileptic hippocampus, entorhinal cortex, and subiculum. *J. Neurosci.* 22 5694–5704. 10.1523/jneurosci.22-13-05694.2002 12097521 PMC6758193

[B118] Stein A. G. Eder H. G. Blum D. E. Drachev A. Fisher R. S. (2000). An automated drug delivery system for focal epilepsy. *Epilepsy Res.* 39 103–114. 10.1016/S0920-1211(99)00107-2 10759298

[B119] Sterman M. B. (2000). Basic concepts and clinical findings in the treatment of seizure disorders with EEG operant conditioning. *Clin. EEG Neurosci.* 31 45–55. 10.1177/155005940003100111 10638352

[B120] Stidd D. A. Vogelsang K. Krahl S. E. Langevin J. P. Fellous J. M. (2013). Amygdala deep brain stimulation is superior to paroxetine treatment in a rat model of posttraumatic stress disorder. *Brain Stimul.* 6 837–844. 10.1016/j.brs.2013.05.008 23835167

[B121] Takeuchi Y. Berényi A. (2020). Oscillotherapeutics-time-targeted interventions in epilepsy and beyond. *Neurosci. Res.* 152 87–107. 10.1016/j.neures.2020.01.002 31954733

[B122] Takeuchi Y. Harangozó M. Pedraza L. Földi T. Kozák G. Li Q. (2021). Closed-loop stimulation of the medial septum terminates epileptic seizures. *Brain* 144 885–908. 10.1093/brain/awaa450 33501929

[B123] Thut G. Miniussi C. Gross J. (2012). The functional importance of rhythmic activity in the brain. *Curr. Biol.* 22 R658–R663. 10.1016/j.cub.2012.06.061 22917517

[B124] Truccolo W. Ahmed O. J. Harrison M. T. Eskandar E. N. Rees Cosgrove G. Madsen J. R. (2014). Neuronal ensemble synchrony during human focal seizures. *J. Neurosci.* 34 9927–9944. 10.1523/JNEUROSCI.4567-13.2014 25057195 PMC4107409

[B125] Truong N. D. Nguyen A. D. Kuhlmann L. Bonyadi M. R. Yang J. Ippolito S. (2018). Convolutional neural networks for seizure prediction using intracranial and scalp electroencephalogram. *Neural Netw.* 105 104–111. 10.1016/j.neunet.2018.04.018 29793128

[B126] Viglione S. S. Walsh G. O. (1975). Proceedings: epileptic seizure prediction. *Electroencephalogr. Clin. Neurophysiol.* 39 435–436. 51767

[B127] Vöröslakos M. Takeuchi Y. Brinyiczki K. Zombori T. Oliva A. Fernández-Ruiz A. (2018). Direct effects of transcranial electric stimulation on brain circuits in rats and humans. *Nat. Commun.* 9:483. 10.1038/s41467-018-02928-3 29396478 PMC5797140

[B128] Wake H. Moorhouse A. J. Jinno S. Kohsaka S. Nabekura J. (2009). Resting microglia directly monitor the functional state of synapses in vivo and determine the fate of ischemic terminals. *J. Neurosci.* 29 3974–3980. 10.1523/JNEUROSCI.4363-08.2009 19339593 PMC6665392

[B129] Walker G. (1931). On periodicity in series of related terms. *Mon. Weather Rev.* 59 277–278.

[B130] Wang Y. Xu C. Xu Z. Ji C. Liang J. Wang Y. (2017). Depolarized GABAergic signaling in subicular microcircuits mediates generalized seizure in temporal lobe epilepsy. *Neuron* 95 92–105.e5. 10.1016/j.neuron.2017.06.004 28648501

[B131] Wyler A. R. Ojemann G. A. Ward A. A. (1982). Neurons in human epileptic cortex: correlation between unit and EEG activity. *Ann. Neurol.* 11 301–308. 10.1002/ana.410110311 7092182

[B132] Yule G. U. (2012). On a method of investigating periodicities in disturbed series, with special reference to wolfer’s sunspot numbers. *Philos. Trans. R. Soc. Lond. A* 226 267–273. 10.1017/cbo9781139170116.013

[B133] Zalay O. C. Bardakjian B. L. (2009). Theta phase precession and phase selectivity: a cognitive device description of neural coding. *J. Neural Eng.* 6:036002. 10.1088/1741-2560/6/3/036002 19436082

[B134] Zalay O. C. Serletis D. Carlen P. L. Bardakjian B. L. (2010). System characterization of neuronal excitability in the hippocampus and its relevance to observed dynamics of spontaneous seizure-like transitions. *J. Neural Eng.* 7:036002. 10.1088/1741-2560/7/3/036002 20404398

[B135] Zrenner C. Desideri D. Belardinelli P. Ziemann U. (2018). Real-time EEG-defined excitability states determine efficacy of TMS-induced plasticity in human motor cortex. *Brain Stimul.* 11 374–389. 10.1016/j.brs.2017.11.016 29191438

